# Changes in microbial composition during flue-cured tobacco aging and their effects on chemical composition: a review

**DOI:** 10.1186/s40643-025-00883-8

**Published:** 2025-05-21

**Authors:** Yangyang Yu, Yongfeng Yang, Tao Jia, Hang Zhou, Yao Qiu, Mengyao Sun, Hongli Chen

**Affiliations:** 1 College of Tobacco Science, Henan Agricultural University, No. 218, Pingan Road, Zhengdong-New District, Zhengzhou, 450002 China; 2 China Tobacco Henan Industrial Co., Ltd, Zhengzhou, 450000 China; 3 Tianchang International Co., Ltd, Xuchang, 461000 China

**Keywords:** Aging, Aroma components, Chemical composition, Microorganism, Tobacco

## Abstract

The aging is a crucial stage in tobacco processing, which contributes to the reduction of impurities and irritation, and the stabilization of the internal chemical composition of the leaves. However, it usually takes a long time (2–3 years) for the nature aging process of tobacco (20 °C–30 °C, relative humidity of 65–75%), which seriously affects the processing efficiency of tobacco. Microorganisms play an important role in the change of chemical composition and characteristic aromatic substances of tobacco. *Acinetobacter*, *Sphingomonas Aspergillus*, *Bacilli*, and *Pseudonocardia* is the main microorganism in the aging process of tobacco, which increasing the aromatic substances (such as alcohols, ketones, and esters) by the action of the enzymes and metabolites, and degrade the harmful components (such as alkaloid, nicotine and nitrosamines in tobacco). This review systematically summarizes recent advancements in understanding the primary microbial composition and the changes in chemical composition during tobacco aging. This knowledge is helpful for screening functional strains, and control the process of tobacco aging by the inoculation of these strains.

**Graphical Abstract:**

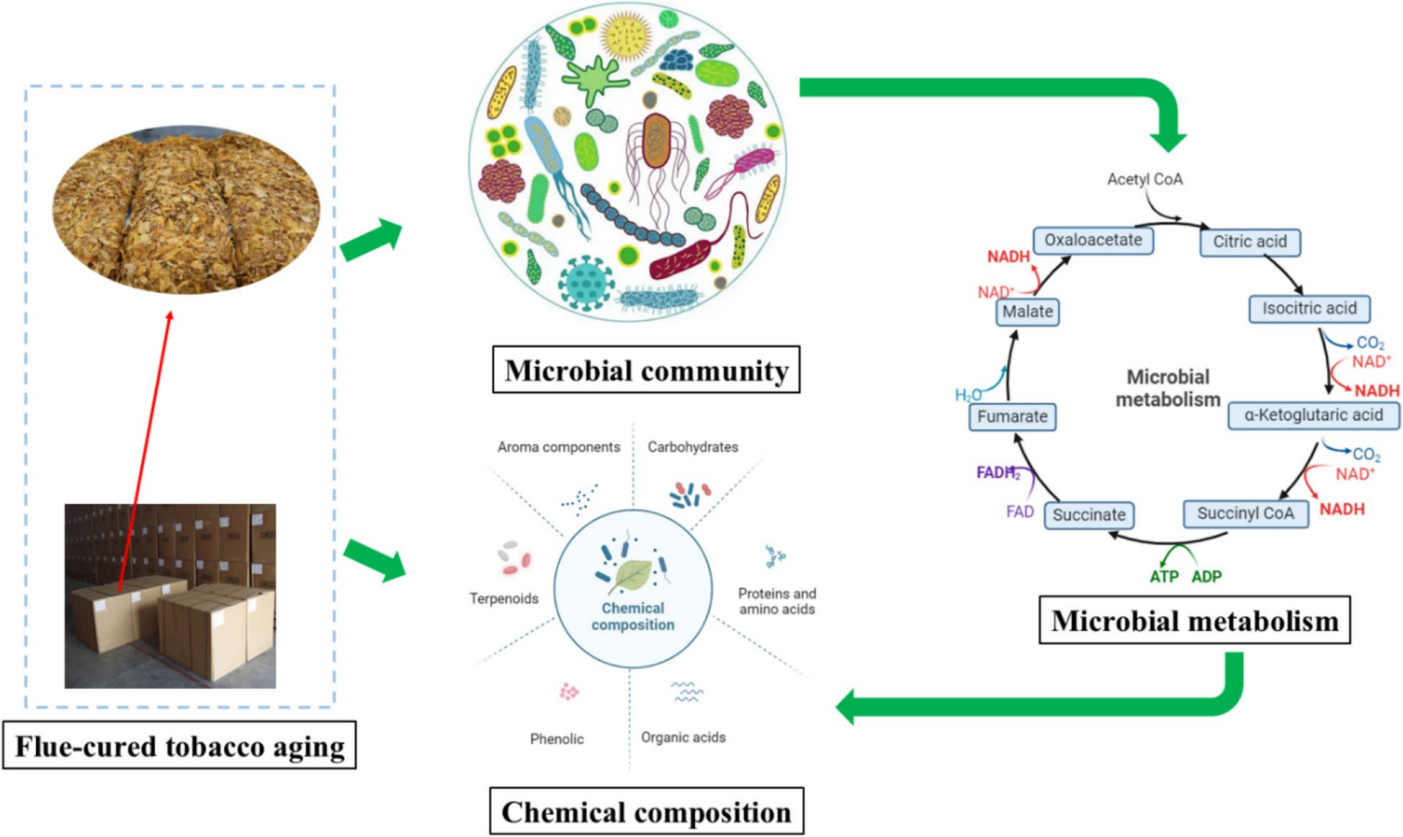

## Introduction

Cigarette products are predominantly derived from tobacco (*Nicotiana tabacum* L.), which is one of the most significant cash crops worldwide (Xu et al. 2019). China is the largest producer and consumer of tobacco in the world. Statistically, 315 million smokers in China consume 44% of the world’s cigarettes. The yearly planting area is about one million ha, and the production yield reaches 1.81 million kg which is about 46.7% of the production in the world (Li et al. 2020). The processing of tobacco involves several key steps, including flue-curing, threshing, redrying, aging, and rolling. In particulars, flue-cured tobacco undergoes a mandatory aging period of 2 years under specific conditions (20 °C–30 °C, relative humidity of 65–75%). This aging process is essential for enhancing the qualities of tobacco (Wu et al. 2022). Unaged tobacco, characterized by quality defects such as intense threshing smoke, green miscellaneous gas, and lack of fragrance, is unsuitable for use in cigarette products (Li et al. 2022). Tobacco aging serves to harmonize the internal chemical composition of tobacco leaves, minimize impurities and irritants, and enhance the smoking quality of tobacco leaves (Yang et al. 2018).

**Fig. 1:**
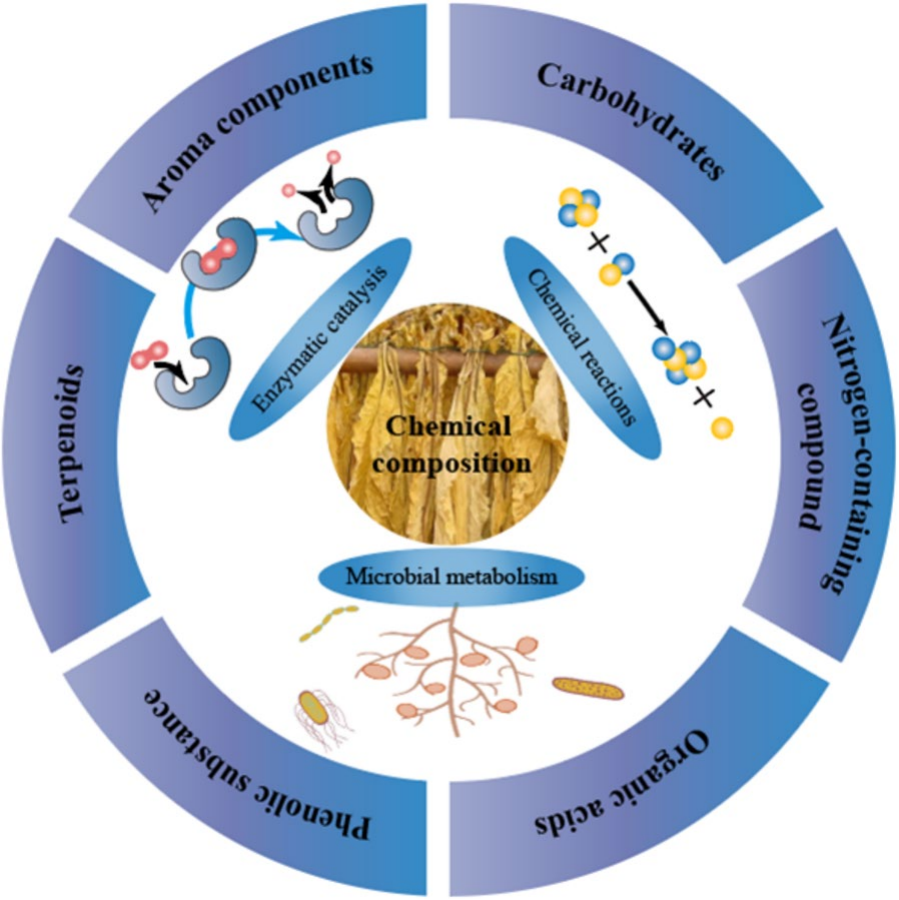
The primary metabolic pathways related to the chemical composition during tobacco aging

Tobacco aging, under specific temperature and humidity conditions, is a process that brings about physical and chemical modifications in tobacco leaves, leading to a significant enhancement in aroma and flavor (Zhou et al. 2020). As tobacco ages, carbohydrates, proteins, and other organic compounds undergo constant decomposition and transformation, including those responsible for tobacco aroma (Banozic et al. 2020). Microbial metabolism (Fig. 1) and oxidation reactions, alongside enzymatic catalysis, play pivotal roles in tobacco aging, as reported (Li et al. 2020). Apart from reducing nicotine content, regulating the coordination of chemical components, and accelerating the aging process, microorganisms also possess the ability to prevent tobacco mildew (Fan et al. 2023). The complex microbial communities present on and within tobacco leaves secrete multiple enzymes that facilitate the degradation of macromolecules (e.g., proteins, starch, cellulose) and harmful compounds (e.g., nicotine) in tobacco leaves while producing flavor components (e.g., terpenes, carbonyls), ultimately contributing to the development of tobacco-specific flavors (Jia et al. 2023). It was reported that *Bacillus subtilis*, *Bacillus circulans*, *Bacillus megaterium*, and *Bacillus thuringiensis* were employed to enhance the development of a desirable aroma and improve the smoking qualities of the tobacco (Wang et al. 2018). Besides, *Pseudomonas spp*. could degrade nicotine and improve the quality of tobacco leaves (Li et al. 2010).

The management of microbiota and its essential enzymes holds promise for enhancing tobacco quality. Dai et al. (2020) screened *Bacillus subtilis* ZIM3 from tobacco, which exhibited high amylase and cellulase activities for efficient biodegradation of starch and cellulose in tobacco, and could be applied for industrial tobacco fermentation. Wu et al. (2022) screened *Aspergillus nidulans* strain F4 could promote the degradation of terpenoids and higher fatty acids to enhance tobacco flavor by secreting highly active lipoxygenase and peroxidase, which verified the effect of tobacco-microbes on tobacco quality. However, the composition and interactions of the active microbial community remain poorly understood due to the intricate nature of microbial communities and metabolic pathways involved in tobacco aging. And, there is still a lack of systematic research on the fermentation mechanism of microbial communities in tobacco fermentation (Jia et al. 2023). Besides, there are few reviews reports on the changes of microorganisms and chemical substances during tobacco fermentation. It is critical to explore the association between the microbes and characteristic metabolites during the aging process, providing guidance for optimizing the tobacco aging process, including controlling process parameters and selecting starter culture (Chattopadhyay et al. 2023). 

Therefore, microorganisms play an important role in regulating of tobacco aging and improving the quality of tobacco leaves during the aging process. A comprehensive understanding of the microbiomes involved in key metabolites is helpful for the improvement in the aging technology of tobacco. To enhance the aging process of tobacco leaves and elevate the overall quality of tobacco, it is imperative to delve into the mechanisms, specifically focusing on the key enzymes and metabolic pathways of essential functional microorganisms during tobacco aging, and their influence on the development of tobacco flavors. The primary objectives of this review are as follows: (1) to elucidate the alterations in the chemical composition of tobacco leaves throughout the aging process; (2) to examine the shifts in microbial populations during the aging of tobacco leaves; and (3) to ascertain the roles played by microorganisms in the aging of tobacco leaves. The insights presented in this review substantiate the theoretical concept that microbial metabolism plays a pivotal role in mediating tobacco aging. This knowledge is helpful to improve tobacco fermentation technology.

## Primary chemical composition in tobacco

**Fig. 2:**
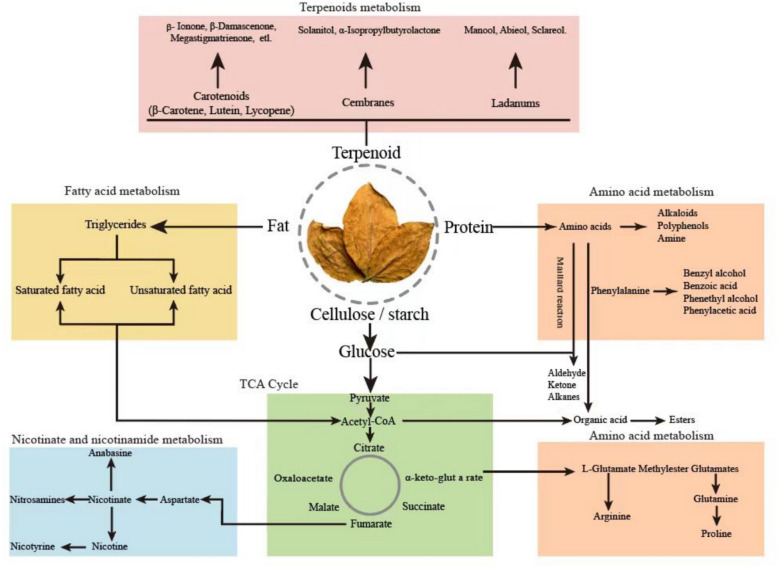
Metabolisms of essential compounds during tobacco aging

When tobacco undergoes aging under suitable environmental conditions, it undergoes internal transformations in its chemical composition, leading to an enhancement in its overall quality. Throughout the aging process, carbohydrates, proteins, and other organic compounds constantly degrade and undergo transformations, as do the compounds responsible for imparting the characteristic aroma (see Fig. 2). After aging, the quality defects will be improved to a certain extent, such as excessive odor and high irritant components. The breakdown of toxic tobacco compounds is closely linked with the metabolism of nicotinate and nicotinamide. The unique flavor profile of tobacco is the result of multiple aromatic compounds and the intricate interactions between them. Previous research has shown that chemical reactions, microbial metabolism, and enzymatic catalysis have the potential to alter the chemical composition of tobacco (refer to Fig. 1) (Banozic et al. 2020). This aging process could reduce the content of macromolecular substances such as protein, starch, and pectin and degrade harmful substances of tobacco leaves, such as organic amines and alkaloids (Wang et al. 2018). It has been found that *Pseudomonas* and *Arthrobacter* bacteria could efficiently degrade nicotine, a toxic compound that seriously affects human health and represents more than 90% of the total alkaloid content of tobacco (Wang et al. 2022). This section provides a comprehensive overview of the alterations that occur in the chemical composition of tobacco during the aging process.

### Carbohydrates

Tobacco leaves have around 50% carbohydrates. Especially, starch (10–30%) and cellulose (0–25%) are essential components of tobacco leaves, which affect the quality of tobacco (Reid et al. 1937). However, when burned, high carbohydrate contents will result in several harmful precursor compounds and an unpleasant charring odor (Dai et al. 2020). Starch affects the completeness and combustion velocity of cut tobacco in a burning cigarette and produces an unpleasant charring odor (Dai et al. 2020). Burning cellulose could degrade it into hydroxymethylfurfural (HMF) and l-glucosan, which has an unpleasant odor (Ning et al. 2023). On the other hand, other research revealed that starch and cellulose might combust and degrade thermally, such as formaldehyde and acetaldehyde, which are regarded as carcinogenic compounds (Banozic et al. 2020). Thus, improving the quality of tobacco leaves requires appropriate degradation of starch and cellulose. Starch and cellulose could be biodegraded into sugars by biological conversion. Sugar, particularly reduced sugar, contributes significantly to the aroma of tobacco and enhances its flavor and aroma, all of which are favorable aspects of tobacco smoking (Roemer et al. 2012). Additionally, these amino acids may react with reduced sugars to form nutty, sweet, and popcorn-flavored Maillard complexes (Hinneh et al. 2018), promoting the formation of the aroma of tobacco.

Carbohydrates provided a carbon source and energy to keep microbes alive on the surface of tobacco, while microorganism could degradate carbohydrates to aroma substances and improve tobacco quality during the natural tobacco aging (Wang et al. 2018). Wu et al. (2022) found that carbohydrate metabolism had the highest relative abundance and increased as aging progresses. Jia (2023) revealed the microbial structure and succession pattern during tobacco aging by high-throughput sequencing, and found that the relative abundances of *Aspergillus*, *Staphylococcus* and *Filobasidium* increased first and then decreased during aging, and occupied the dominant position of bacterial and fungal communities on the 21st day, respectively. It was reported that *Aspergillus*, *Staphylococcus* and *Filobasidium* could produce enzymes necessary for starch degradation, i.e., with debranching activity α-Amylase and glucoamylase, thereby leading to increases of total and reducing sugar contents and providing precursor materials for subsequent growth of other microorganisms (Jia et al. 2020a; Lakshmi et al. 2013). Ning et al. (2023) isolated *Bacillus* subtilis B1 with high protease, amylase, and cellulase from the first-cured tobacco followed by using them for solid-state fermentation of tobacco. Results showed that strains *Bacillus* B1 could promote the degradation of nicotine, starch, and protein of tobacco, leading to increasing reducing sugars. Moreover, the tobacco sensory quality was significantly improved. Dai et al. (2020) isolated *Bacillus* subtilis ZIM3 from the aging flue-cured tobacco leaves, which exhibited high amylase and cellulase activities for efficient biodegradation of starch and cellulose in tobacco.

### Nitrogen-containing compounds

Nitrogen-containing components found in tobacco products encompass proteins, free amino acids, alkaloids, nitrates, N-nitrosamines, and others, which influence on the quality of tobacco. Generally, nitrogen compounds constitute approximately 15% of the dry weight of tobacco. A high concentration of nitrogen compounds can give rise to pungent irritation, overbearing impurities, and a bitter taste in the smoke. Conversely, a low concentration can lead to a lack of intensity and strength in the smoke, as well as insufficient aroma (Gao et al. 2014). Nicotine provides kick and adds flavor, but in high amounts can be irritating; Protein can increase the power of smoke, and bitter taste; Amino acids are aroma precursors of tobacco leaves and precursors of Maillard reactants, which make important contributions to tobacco aroma, but their high content will make aroma quality deteriorate, irritation increase and impurity increase.

Nitrogenous compounds are vital sources of nutrition for the tobacco microorganisms. Wang et al. (2018) investigate the surface bacterial communities and their functional diversity from aged tobacco by 16S rRNA sequencing and PICRUSt software, and found abundant nitrogen metabolism, including several involved in the biosynthesis of flavors and fragrances and the degradation of harmful compounds, such as nicotine and nitrite. After aging, the content of nitrogen compounds in tobacco leaves decreased by more than 33%. Microorganisms degrade nitrogenous compounds like protein, nicotine to aroma substances during tobacco aging, and further improve the taste and smoking quality of tobacco. Previous studies showed that *Bacillales* and *Candida* degrade nitrogenous substrates and synthesize flavor substances, while *Candida* also contribute to maintaining the stability of microbial community (Zhou et al. 2020; Jia et al. 2023). In addition, some nitrogenous compounds are decomposed into ammonia and lost to the air, resulting in a reduction in the total amount of nitrogen compounds (Liu et al. 2015).

#### Proteins and amino acids

Protein is the primary macromolecular nutrient in tobacco, making up 8–15% of the dry weight. Proteins provide the skeleton structure of tobacco, but protein is also the precursor of several toxic compounds in tobacco, affecting the quality of tobacco. For example, tobacco protein could produce the throat choking and charred feather smell, such as quinoline, cyanic acid, and benzopyrene with cigarettes smoking (Wu et al. 2021). With the tobacco age, the soluble protein contents decreased by 12% to 18% and the extent of aging of the tobacco was characterised by determinations of the contents of cytosolic proteins (Mytinova et al. 2011). Usually, tobacco protein could be degraded into amino acids during the aging. These incredibly aromatic amino acids could then be converted into several aromatic compounds (e.g., benzene acetaldehyde, 2-methyl-benzaldehyde, 3-amino benzoic acid, and phenyl ethenone) and esterified to methyl phenyl acetate and methyl benzoate (Arihara et al. 2017).

**Fig. 3:**
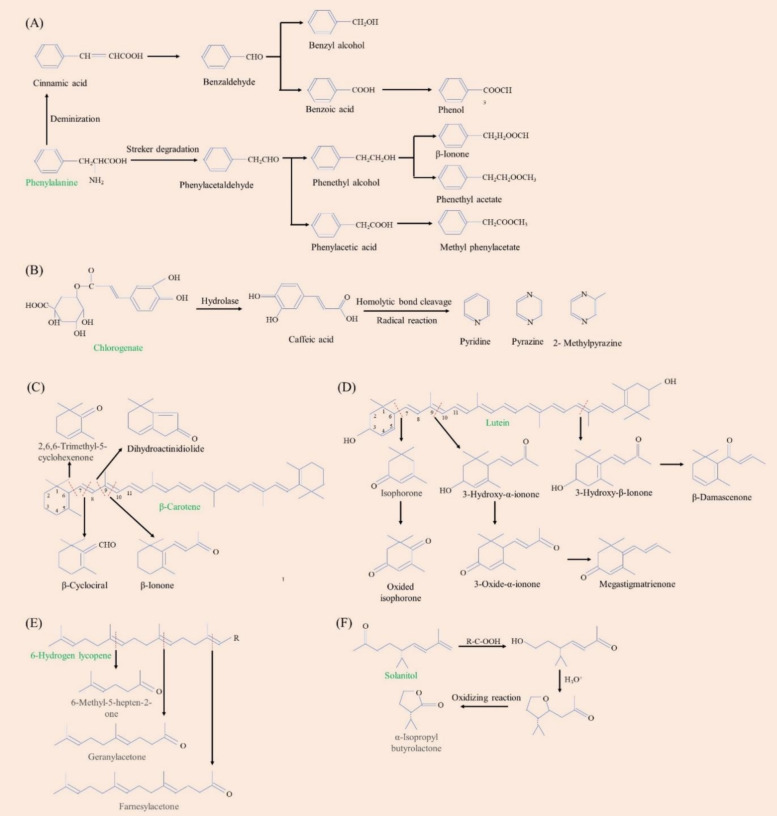
The degradation of key compounds during tobacco aging. **A** phenylalanine; **B** chlorogenate; **C** β-Carotene; **D** Lutein; **E** Lycopene; and **F** Solanitol

Amino acids are the basic building blocks of proteins, and they play a role in regulating the flavor and aroma of tobacco. Tobacco contains over 30 different types of amino acids, the majority of which are in a free form, with elevated levels of proline and aspartic acid. Free amino acids can interact with sugars produced through the biodegradation of starch and cellulose, contributing to the development of tobacco aroma. Amino acids can combine with reducing sugars through the Maillard reaction to produce new compounds, resulting in reduced amino acid content. There were many kinds of Maillard reaction products with unique aroma, including aldehydes, ketones, alkanes, *N*-substituted glycosylamines, nitrogenous heterocyclic compounds, and Amadori compounds (Chen et al. 2019). Additionally, free amino acids serve as precursors for alkaloids and polyphenols, participating in the carbon and nitrogen metabolism that influences the formation of nitrogen-containing compounds and other chemical compounds. Phenylalanine metabolism is a crucial metabolic pathway in tobacco, leading to an increasing trend in compounds such as phenylcarbinol, phenylethanol, benzaldehyde, and phenylacetaldehyde during the aging process (see Fig. 3A) (Meng et al. 2022; Zou et al. 2023).

#### Nitrates and nitrosamines

Nitrates and tobacco-specific nitrosamines (TSNAs) are other nitrogen-containing compounds found in tobacco (Wei et al. 2014). Nitroso-nicotinine, N-nitroso-nicotinine, nitroso-nitrosamine, and 4-methylnitrosamine-1-3-pyridine-1-butanone are the major nitrogen oxides that are formed during the nitrosation of tobacco alkaloids, which includes nicotine, norotinoids, and neonicotinoids. Undesirable byproducts of the fermentation process, known as TSNAs, are a significant source of cancer in tobacco (Li et al. 2020; Sarlak et al. 2020). Previous studies have shown a positive correlation between the concentration of nitrates during tobacco fermentation and the accumulation of TSNAs (Lawler et al. 2013). According to reports, nitrate is first reduced to nitrite, which then reacts with alkaloids to form TSNAs. This process is thought to be carried out by the metabolism of nitrate- and nitrite-reducing bacteria (Atawodi and Richter 2016; Rivera and Tyx 2021), such as *Bacillus*, *staphylococcus*, *corynebacterium*, etc. (Fisher et al. 2012; Giacomo et al. 2007). To improve the quality of tobacco leaves, it is important to decrease the accumulation of nitrite and nitrosamines during tobacco aging process. Wei et al. (2014) isolated a *Bacillus amylolytica* DA9 strain that exhibited strong nitrite reducing capacities and found that the strain decreased TSNA content by reducing the nitrite precursor content of TSNA. To effectively avoid the accumulation of nitrites and nitrification in tobacco leaves, Vigliotta et al. isolated and screened the yeast strain *Debaryomyces hansenii* TOB-Y7 from tobacco leaves at the early stage of tobacco maturation and applied it to the fermentation cigar tobacco (Vigliotta et al. 2007).

#### Nicotine

The content of alkaloids in tobacco varies greatly, with low ones containing less than 0.5% and high ones up to more than 10% (Ning et al. 2023). The existence of tobacco alkaloids is the main sign that tobacco is different from other plants. Alkaloids and their salts in tobacco have a strong hydration effect and can be volatilized with water vapor under acidic reaction conditions. Although the amount of free nicotine volatilized in this way is not large, it has a great impact on the smoking quality of tobacco leaves, and it is easy to make the finished products absorb spicy and choking. It is worth noting that tobacco harbors more than 20 distinct types of alkaloids (Li et al. 2020). In the realm of tobacco, nicotine is the most prevalent alkaloid, accounting for over 95% of the total alkaloid content (Zhong et al. 2010). Tobacco used to make cigarettes usually contain less than 2% nicotine. Nicotine primarily exists in the form of organic acid salt binding state within tobacco, but when exposed to high temperatures, it undergoes a conversion into a free state. This transformation leads to the formation of pyridine, which plays a crucial role in generating the characteristic aroma associated with tobacco (Weeks et al. 1995). By incorporating an appropriate amount of nicotine, manufacturers can enhance the flavor of cigarettes. However, the elevated nicotine levels in tobacco come with health hazards, as they have the potential to induce addiction, toxicosis, and ultimately facilitate the conversion of nitrosamines (Wu et al. 2022). The widely applied strategy for reducing nicotine in cigarettes is mixing high nicotine content tobacco with low-nicotine ones (Zhao et al. 2012). Numerous studies have demonstrated nicotine degradation by microbes (Zhong et al. 2010).

The aging process of tobacco decreased the harmful effects of tobacco on humans, including nicotine, anatabine, and nornicotine. During to tobacco aging, microorganisms are crucial to the degradation of nicotine. Microorganisms such as *Candida* (Jia et al. 2023), *Pseudomonas* (Mu et al. 2020), *Agrobacterium* (Wang et al. 2009), *Arthrobacter*, *Rhodococcus* (Gong et al. 2009), and *Cellulomonas* (Law et al. 2016) have been reported to be capable of degrading nicotine. Additionally, researcher has revealed that some actinomycetes and fungi are capable of degrading nicotine. Jia et al. (2023) isolated two *Candida* strains (*C. parapsilosis* and *C. metapsilosis*) from tobacco leaves and assessed their ability to ferment tobacco. The results showed that *C. parapsilosis* and *C. metapsilosis* bioaugmentation could shorten the fermentation cycle, enhance flavor, reduce total nitrogen and alkaloids in cigar tobacco leaves to increase safety. Several microorganisms in the fermentation process can effectively degrade nicotine, according to these findings (Ruan et al. 2019). However, strains with stronger abilities to degrade nicotine suitable for application in tobacco are also still required.

### Organic acids

Organic acids in tobacco leaves are divided into non-volatile organic acids (citric acid, malic acid, oxalic acid, oleic acid, and succinic acid) and volatile organic acids. Non-volatile organic acids have the ability to modify a variety of tobacco leaf properties, including pH salt synthesis with alkaloids, free nicotine content, mellow flue gas, and enhance leaf sensory qualities. However, volatile organic acids may enter the flue gas during the tobacco smoke suction process to modify its pH and enhance the quality and concentration of tobacco leaves. About 70%–80% of organic acid is non-volatile, significantly impacting the sensory quality of cigars (Hu et al. 2022). While oxalic acid and citric acid can adversely affect the taste of tobacco, malic acid has been shown to improve the roundness of tobacco and reduce irritation (Popova et al. 2019). Lactic acid can lessen discomfort, improve the aftertaste, and make tobacco smoke seem finer and rounder. Microbial metabolism and the change in organic acid content in tobacco aging were closely related. According to reports, as tobacco ages, its overall volatile acid content increases, and its concentration of β-methyl valeric acid and isovaleric acid significantly increases. In contrast, its concentrations of butyric acid and propionic acid decreased (Xuefeng et al. 2017), which could be produced by esterification with alcohols.

### Polyphenols

**Fig. 4:**
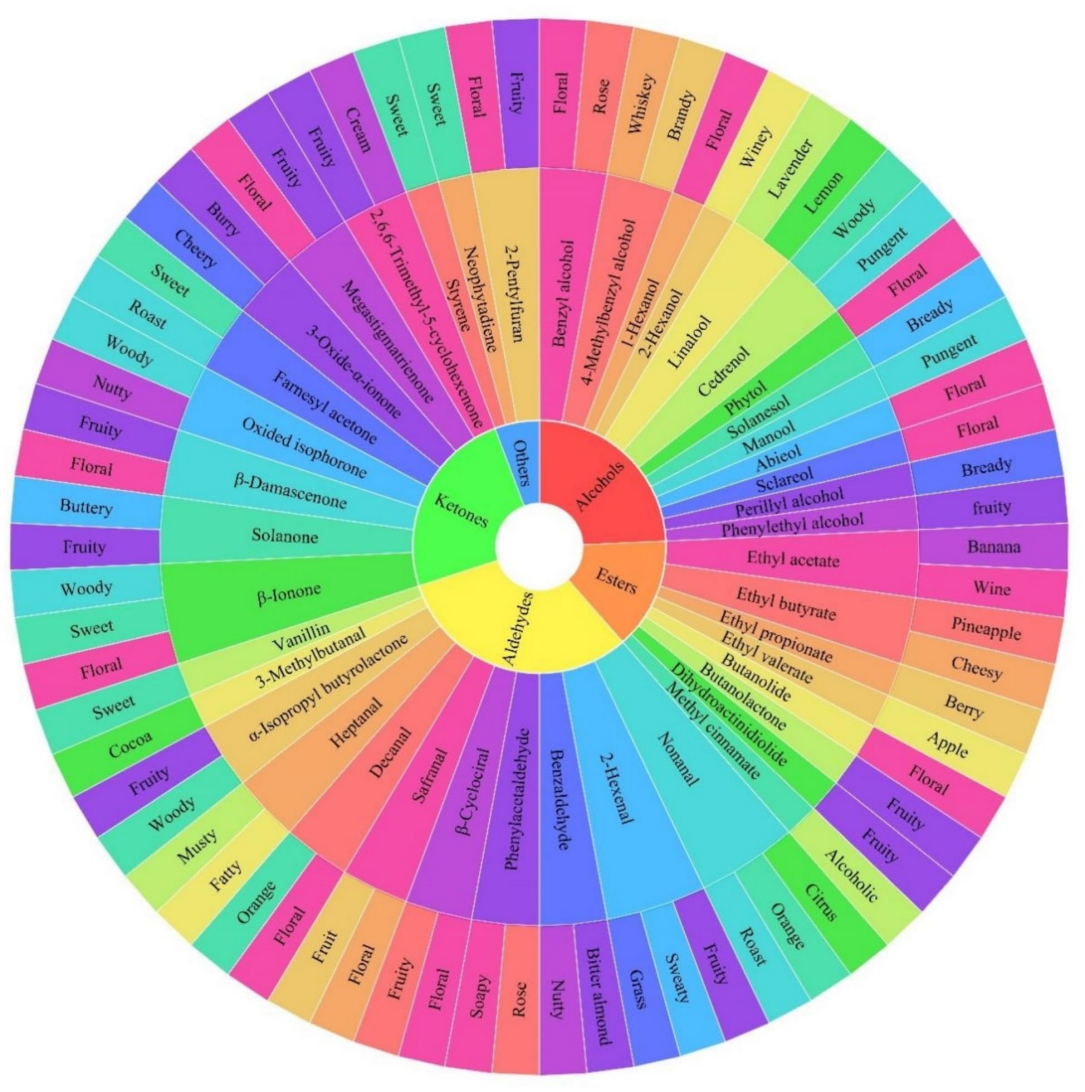
Tobacco aroma components and their corresponding aroma descriptors. This figure was created according to previous studies (Wu et al. 2022; Li et al. 2022; Fan et al. 2023; Zou et al. 2023) and http://www.thegoodscentscompany.com/search2.html

Tobacco is rich in polyphenols, which make up 1.8–5.0% of its dry weight. The majority of these polyphenols are produced during the baking process when they pyrolyze with other macromolecular components. The quality of flue-cured tobacco is mainly determined by polyphenols, precisely the color and aroma of the flue-cured leaves and the odor and taste of the smoke. The sensory quality of tobacco leaves is impacted by the precipitate of the polyphenol and protein combination, which astringently increases the sensation of bitterness and dryness in the mouth (Ramos-Pineda et al. 2020). The polyphenol and flue gas vigor and concentration are also related. The flue gas becomes more bitter and irritating when the polyphenol concentration is too high, and the tobacco leaves become weak when the concentration is too low (Zou et al. 2023). Quinone was readily produced from polyphenols using polyphenol oxidase and peroxidase. Cured leaf browning is caused by the interaction of quinone with amino acids to form a brown substance. The primary polyphenols found in tobacco are tannins (chlorogenic acid, cryptochlorogenic acid, neochlorogenic acid, etc.), flavonoids (rutin, camphenol-3-rutin, etc.), and coumarins (scopolamine, etc.). Three main phenolic compounds—chlorogenic acid, rutin, and scopolamine—affect the color, aroma, and absorption of cigarettes in significant ways (Meng et al. 2022). The degradation of chlorogenic acid can result into pyridine, pyrazine, and 2-methylpyrazine (Fig. 3B), which have a nutty and roasted flavors for tobacco leaves (Fig. 4).

Tobacco aging causes polyphenols to undergo further degradation and transformation to balance their content, which mellows the aroma of tobacco and reduces irritation (Wu et al. 2013). Microorganisms create polyphenol oxidase and peroxidase, which can degrade polyphenols into smaller molecules. These chemicals can then combine with amino acids and other macromolecular compounds to increase the contents of Maillard reaction products and aromatic amino acid degradation products (Zou et al. 2021). Permeability, aroma, impurity, and aftertaste of tobacco were found to be significantly correlated with polyphenolic substances, while the concentrations of phenolic substances were found to decrease with an increase in tobacco aging time. Feng et al. (2019) applied *Sphingosinomonas* to age tobacco, which significantly improved the ability to degrade chlorogenic acid and rutin in tobacco and improve the sensory quality of tobacco leaves.

### Terpenoids

Carotenoids, ladanums, and cembranes are the primary components in the class of molecules known as terpenoids, which have an isopentylene structure and are significant precursors of aroma in tobacco. The general structure of carotenoids, which are compounds or oxidized derivatives with eight isoprene groups attached to the head and tailas shown in Fig. 3.

The findings of the smoking evaluation benefit from the carotenoids in tobacco. Tobacco contains carotenoids mostly in the form of cyclic β-carotene, non-cyclic lutein, and lycopene. A variety of C9–C13 compounds, including β-cyclocitral, β-ionone, 2,6,6-Trimethyl-5-cyclohexenone, and dihydroactinidiolide, were formed when β-carotene underwent oxygenation at various carbon bond positions during the aging process of tobacco (Fig. 3C). A small amount of dihydroactinidiolide can reduce smoking irritation, while ionone can increase the floral aroma of tobacco. Figure 3D illustrates the degradation and transformation process of lutein. At positions 9–10, lutein breaks the double bond to form 3-hydroxy-α-ionone. It can then undergo a redox proven to convert 3-oxide-α-ionone. Next, a dehydration reaction converted 3-oxide-α-ionone into megastigmatrienone. Megastigmatrienone is a particularly significant tobacco flavoring ingredient that can increase the floral and woody notes of flue-cured tobacco (Wu et al. 2022). When the carbon chain breaks at the 6–7 position of lutein, oxidized isophorone—a tobacco flavor that is widely used—is produced. Furthermore, lutein produces 3-hydroxy-β-ionone by breaking the double bond at positions 21–22, which can then be converted into β-Damascenone. The distinct rose aroma of β-Damascenone can improve the aroma quality of tobacco leaves. Lycopene is degraded into 6-methyl-5-hepten-2-one, geranylacetone, and farnesylacetone during aging (Fig. 3E).

The unique aroma components of aromatic tobacco, such as manool, abieol, sclareol, etc., are called ladanums compounds. Solanitol, the degradation product of cembranes compounds, is an important flavor component in tobacco leaves. Solanitol has the ability to be converted into α-isopropyl butyrolactone (Fig. 3F), a unique tobacco aroma component.

Research has focused on the late mellow period of tobacco leaves since it is when the content of terpenoid breakdown products increases and can give the flue gas a sweet and fragrant appearance. During the process of natural aging, the content of products of carotenoid degradation primarily increased. We compared the terpene degradation products present in two kinds of flavor tobacco leaves. It was found that compared to Luzhou-flavor tobacco leaves, the total amount of terpene degradation products in clear flavor tobacco leaves was significantly higher (Gao et al. 2014).

### Aroma components

The primary component affecting the flavor and industrial availability of tobacco, and the evaluation indexes of tobacco quality, was the odorant compounds. The aging process of tobacco led to a significant improvement in its aroma quality, which was strongly associated with the transformation and accumulation of aroma components. Based on their functional groups, odorant compounds in tobacco can be divided into aldehydes, ketones, alcohols, esters, and heterocyclic compounds. Similar odorant properties can be produced by chemicals in the same class and odorant group (Fig. 4).

Alcohols are derived from the degradation of glucose and amino acids during tobacco aging, mainly including ethanol and higher alcohols. Alcohols often showed an increasing trend during tobacco aging (Zhang et al. 2024). Fermented tobacco contains various alcohol compounds, including benzyl alcohol, phenylethyl alcoho, 1-hexano, perillyl alcoho, 4-methylbenzyl alcohol(Zou et al. 2023), fatty alcohols, alicyclic alcohols, aromatic alcohols, sterols, and terpene alcohols (Wu et al. 2022; Zou et al. 2023). Strong perfuming group like the hydroxyl group are associated with unsaturated bonds to enhance its aroma. Alcohols with 7–10 carbon atoms often have a pleasant aroma, whereas alcohols with more than 10 carbon atoms will eventually become odorless. Through the glycolytic pathway and pyruvate metabolism, yeast and lactic acid bacteria (LAB) are the primary producers of ethanol, which has a wine-like flavor (Xuefeng et al. 2017). Tobacco leaves are scented with floral and fruity aromas thanks to the metabolism of phenylethyl alcohol and benzoyl alcohol, which are produced during the fermentation of phenylalanine (Fan et al. 2023). In addition, 1-Hexanol is a significant volatile that gives tobacco their grassy, fishy aroma (Li et al. 2022).

In addition, most esters provide flavor to a variety of foods by having a pleasant fruit or wine aroma (Dan et al. 2020). The production of esters, such as ethyl acetate (banana smell), ethyl propionate, ethyl valerate (sweet apple smell), ethyl butyrate (pineapple smell), butanolide (floral smell), coumarin (floral smell), butanolactone (fruity smell), and dihydroactinidiolide (fruity smell), is primarily carried out by microbial esterases and through non-enzymatic esterification of alcohols and organic acids during tobacco aging (Fig. 4). Most esters can impart sweet and fruity flavors (Yulin et al. 2021). Dihydroactinidiolide may improve the palatability of cigarettes by masking unpleasant flavors (Lv et al. 2012).

Aldehydes have been demonstrated to significantly enhance the flavor of tobacco due to their low odor threshold. They also contribute nutty and fruity aromatic attributes to soy-based fermented foods, primarily through the Strecker degradation process during aging (Ni et al. 2018). This process involves the formation of Strecker aldehydes, such as the degradation reaction of benzaldehyde, phenylacetaldehyde, and 3-methylbutanal. As illustrated in Fig. 4, phenylacetaldehyde emits strong rose floral and soapy aromas, while benzaldehyde imparts bitter almond and nut aromas (Zou et al. 2023). Under acidic conditions, the rearranged sugars suffer cyclization to produce oxygen-containing heterocyclic compounds (such as furfurals) (Wang et al. 2023). Reports indicate that furfural can be generated during the fermentation of tobacco through the Maillard reaction with amino sugars (Wahlberg et al. 1977). Furfural, a common byproduct of this reaction, significantly influences the flavor and color of tobacco (Li et al. 2022; Yuxin et al. 2023). Moreover, the degradation products of carotenoid β-cyclociral contribute fruity and floral aromas. Safranal, which enhances the sweetness and aftertaste of smoke, is also produced during the mellowing process of tobacco leaves, imparting a pleasant flowery and fruity aroma (Li et al. 2022) (refer to Fig. 4).

The primary source of ketones in fermented fish products is the degradation and conversion of terpenoids, such as β-ionone, 2,6,6-trimethyl-5-cyclohexenone, β-damascenone, oxidized isophorone, 3-oxide-α-ionone, and megastigmatrienone (Ni et al. 2018). Hu et al. (2004) have documented an increase in ketones during the natural aging process of tobacco. Megastigmatrienone, also known as “tabanone” was found to be the critical flavor compound in tobacco that gave it a unique flavor and enhanced the smoke concentration (Slaghenaufi et al. 2016). Worldwide use is made of oxidized isophorone, a tobacco spice with notes of roasted wood and nuts. In addition, the floral aroma can be enhanced by β-ionone and β-damascenone (Fig. 4).

Through Maillard reaction, which is highly complex, sugars and amino acids can produce rich aroma substances. Generally, acidic conditions to produce furan aroma substances, while alkaline conditions produce aldehydes, ketones and phenols, as well as common dihydrofurfurone furfural, furfuryl alcohol and pyrrole. Phenylalanine breakdown mainly yields benzaldehyde, phenylethanol and phenylacetaldehyde as aroma substances (Wu et al. 2022). High temperatures can cause alkaloids to pyrolyze into heterocyclic compounds like nicotine, which can then pyrolyze into 3-vinylpyridine during the process of combustion and absorption. Furthermore, it has been shown that after six months of fermentation—roughly ten times that of green tobacco leaves—the amount of neophytadiene produced by the breakdown of chlorophyll during tobacco aging reaches its maximum (Wang et al. 2020). The concentration of neophytadiene then steadily drops with continued aging, possibly as a result of conversion to plant furan and other compounds (Wahlberg et al. 1977).

### Harmful substances in tobacco

Tobacco smoking is the world’s leading cause of avoidable premature mortality. Tobacco and tobacco smoke contain a complex mixture of over 9500 chemical compounds, many of which have been recognized as hazardous to human health by regulatory agencies (Li and Hecht 2022). There are multiple compounds that cause human health problems other than cancer Table 1. The reduction of harmful chemical components within the tobacco leaf is considered as a critical objective in tobacco breeding initiatives.

1. Nicotine

**Table 1:** Harmful substances in tobacco and their effects on health

Harmful substances	Characteristic	Effects on health
Nicotine	The pungent smell of smoke	It's addictive and causes blood vessels to constrict
Tar	Black viscous substance	Contains a variety of carcinogens, increasing the risk of cancer
Carbon monoxide	Colorless and odorless gas	It binds to hemoglobin, resulting in insufficient oxygen to the heart
Nitrosamines	Contains the –N–N=O group	Increasing the risk of cancer
Others	Heavy Metals	Cause harm to human health

Nicotine is a colorless, transparent, oil-like volatile liquid with a stimulating smoke odor. It can act on the brain of smokers, make smokers dependent on tobacco, and is the main component leading to tobacco addiction in two novel nicotine pouch products in comparison with regular smokeless tobacco products and pharmaceutical nicotine replacement therapy products (Li et al. 2024). The direct effect of nicotine on the human brain is to make the smoker feel alert, excited and/or relaxed. Nicotine can also cause blood vessels to constrict, raising blood pressure. The intima of blood vessels is damaged, causing coronary artery spasm, angina pectoris and myocardial infarction (Zhu et al. 2024).

(2) Tar

Tar, sticky substance produced when tobacco is burned. It is present in the smoke in the form of fine particles. Tar contains benzopyrene, benzodiazine and other high carcinogens, and can be attached to the smoker's trachea, bronchus and alveolar surface, produce physical and chemical stimulation, damage the respiratory function of the human body, is the main cause of lung cancer and throat cancer (Li and Hecht 2022). In addition, tar is also the cause of the yellow fingers and teeth of smokers (Oldham et al. 2014).

(3) Carbon monoxide

Carbon monoxide is a colorless and odorless gas, and each cigarette can produce about 20 to 30 ml of carbon monoxide after combustion. It has a 260 times higher affinity with hemoglobin than oxygen and can disrupt the function of blood to deliver oxygen, thus affecting organs throughout the body (Wiseman et al. 2016). Carbon monoxide can promote the increase of cholesterol storage and accelerate atherosclerosis. Substances such as nitric oxide and vapor phase free radicals in carbon monoxide and smoke can also damage vascular endothelial function, increase blood viscosity, promote thrombosis, increase oxidative stress and inflammatory response, and induce or exacerbate cardiovascular diseases.

(4) Nitrosamines

Nitrate is reduced to nitrous acid under the action of microorganisms during the modulation and aging process of tobacco leaves, and then nitrosation reaction with alkaloids produces nitrosamines (Wang et al. 2017). Nitrosamines are a class of *N*-nitrosamine compounds present in tobacco leaves and smoke, which can cause lung cancer, adenocarcinoma and esophageal cancer in animals, and the International Organization for Research on Cancer (IARC) lists it as the highest level of Class I carcinogens. The nitrate content in tobacco showed a significant linear correlation with nitrosamine accumulation (Dong et al. 2024).

(5) Others

When tobacco leaves are burned, cellulose and protein are cracked at high temperature to produce formaldehyde, hydrocyanic acid, acrolein and other toxic substances, which can seriously damage the bronchial mucosa and cause bronchial and lung infection (Oldham et al. 2014). In addition, tobacco absorbs cadmium, mercury, lead, arsenic and other harmful metals from the soil. Into the reproductive system, can cause infertility. Enter the bone, cause bone decalcification, easy to fracture (Dong et al. 2024).

## The microbiota in tobacco aging

### Changes in microorganisms during tobacco aging

Microorganisms could decomposition macromolecules (such as protein, starch, and cellulose) of tobacco through the action of the enzymes and metabolites produced by themselves, increasing the aromatic substances (such as alcohols, ketones, and esters) (Zhou et al. 2020). Meanwhile, microorganisms that can degrade the harmful components, such as alkaloid, nicotine and nitrosamines in tobacco, improving the quality characteristics.

To study microbial diversity in fermented tobacco, both culture-dependent and culture-independent methods can be used. Culture-dependent are time-consuming and limited to culturable microorganisms, which may lead to incomplete information in fermented tobacco (Liu et al. 2023). In the case of culture-independent assays, the total DNA of microorganisms present in fermented tobacco sample can be extracted and analyzed using metagenomics or full length 16S rDNA and 18S rDNA gene sequencing (Chen et al. 2017). It could decipher the structure of the whole microbial community and identify the metabolic capability of such a system (Yu et al. 2022). Wu et al. (2022) found that *Pseudomona*, *Rhizobiale*, and *Bacillales*, comprised the core bacterial microbiome by 16S rRNA gene sequence analysis. And these core microbiomes have a substantial potential for the carbon cycle, amino acid metabolism, aromatic compound metabolism, chitinolysis, cellulolysis, and xylanolysis, and so on. There were changes in the structure of the microbial community in tobacco leaves during their aging process. This might result from antagonism between microorganisms or from the low water content inhibiting the growth of tobacco leaves during the aging process (Zhao et al. 2007). In the early stages, fermented tobacco was used to extract culturable microorganisms such as bacteria, mold, yeast, and actinomyces. The most common numbers and types of these microorganisms were bacteria, including *Bacillus*, *Lactobacillus*, *Clostridium*, *Bacillus*, and *coccus*. The predominant molds were *Aspergillus*, *Rhizopus*, and *Penicillium*; actinomyces and yeasts were sparse. However, the microorganisms that can be cultured in tobacco are the only ones that can be studied using the traditional isolation culture method (Liu et al. 2021). To analyze the variety of the microbial community in tobacco gene-level culture-free technology has been used recently. This method can be more comprehensively reflect the microbial diversity and community structure in the environment (Wang et al. 2015). Jia et al. (2023) identified the core microbiome of 15 tobacco samples using 16S rDNA and ITS amplicon sequencing methods. According to the findings, the dominant bacterial genera were *Staphylococcus*, *Pseudomonas*, *Pantoea*, *Sphingomonas*, *Enterobacter*, *Kosakonia*, and *Methylbacterium*, and the dominant fungal genera were *Aspergillus*, *Alternaria*, *Wallemia*, *Cladosporium*, *Penicillium*, and *Stemphyllium*. Liu et al. (2021) analyzed the microbial communities in tobacco pre- and post-fermentation using Illumina Miseq high-throughput sequencing technology. They discovered that while the relative abundances of *Ralstonia* and *Pseudomonas* increased, and those of *Methylobacterium*, *Pseudonocardia*, *Aureimonas*, *Planococcus*, and *Nocardiopsis* decreased, whereas the relative abundances of *Aspergillus* and *Phaeosphaeriaceae* in the fungal community significantly increased, and those of *Cladosporium* significantly decreased (Giacomo et al. 2007) suggesting the composition of microorganisms changes constantly during tobacco fermentation. Similarly, Li et al. (2020) used high-throughput sequencing to analyze the changes in the microbial communities of tobacco aging and discovered that *Pseudomonas* increased with the increase in fermentation time, whereas *Sphingomonas* and *Methylobacterium* decreased. However, Di et al. (2007) revealed that *Staphylococcus* and *Lactobacillales* were abundantly found in bacteria in the early phase of fermentation, whereas *Actinomycetales* significantly expanded during the late phase. The main microorganisms involved in tobacco fermentation are shown in Table 2. The well-known bacterial populations found in tobacco include *Acinetobacter*, *Bacillus*, *Pantoea*, *Pseudomonas*, *Staphylococcus*, *Stenotrophomonas*, *Sphingomonas*, and *Jeotgalicoccus*, whereas the fungal populations include *Aspergillus*, *Alternaria*, *Cladosporium*, *Cladosporium*, and *Rhizopus*.

**Table 2:** The composition of microbiota and their roles in tobacco fermentation

	Microorganism	Function	References
Bacteria	*Acinetobacter*		Ye et al. (2017)
	*Bacillus*	Degradation of starch, protein, TSNAs, and nicotine	Dai et al. (2020), Wei et al. (2014)
	*Pantoea*	Increasing lipopolysaccharide	Su et al. (2011)
	*Pseudomonas*	Degradation of nicotine	Mu et al. (2020)
	*Staphylococcus*	Degradation of starch and TSNAs	Lakshmi et al. (2013), Fisher et al. (2012), Giacomo et al. (2007)
	*Stenotrophomonas*		Chopyk et al. (2017)
	*Sphingomonas*	Degradation of nicotine	Feng et al. (2019)
	*Jeotgalicoccus*	Degradation of sugars and organic acids	Giacomo et al. (2007)
	*Corynebacterium*	Degradation of TSNAs	Fisher et al. (2012), Giacomo et al. (2007)
	*Debaryomyces*	Degradation of TSNAs	Vigliotta et al. (2007)
	*Agrobacterium*	Degradation of Nicotine	Wang et al. (2009)
	*Arthrobacter*	Degradation of Nicotine	Ruan et al. (2018)
	*Cellulomonas*	Degradation of Nicotine	Law et al. (2016)
Fungal	*Aspergillus*	Degradation of starch	Jia et al. (2020b)
		Degradation of terpenoids; Increasing fatty acids,	Wu et al. (2022)
	*Candida*	nicotine, chlorophyll, and carotenoid degradation	Ruan et al. (2019)
		Degradation of total nitrogen, nicotine, and alkaloids	Jia et al. (2023)
	*Alternaria*		Espinoza-Sánchez et al. (2015)
	*Chaetomium*		Zhou et al. (2020)
	*Cladosporium*		Zhou et al. (2020)
	*Rhizopus*		Wu et al. (2022), Zhou et al. (2020)

### The functions of microorganisms in tobacco fermentation

Microorganisms play a crucial role in the aging process of tobacco by increasing the production of aromatic compounds and reducing the levels of toxic compounds through the activity of metabolites and enzymes (Liu et al. 2015; Wei et al. 2014). In 1953, Tamayo et al. (1953) conducted an experiment aimed at enhancing the aromatic properties of tobacco leaves through microbial inoculation. The findings of this study indicated that the use of *Bacillus* and *micrococcus* strains could effectively enhance the aroma of tobacco leaves. Subsequent research has further demonstrated that the careful selection of appropriate microorganisms and the establishment of favorable fermentation conditions during the mellow fermentation process can significantly enhance the quality and aroma of tobacco leaves.

Table 2 provides an overview of the types and functions of microorganisms. According to reports, that the dominant microorganisms were *Acinetobacter*, *Sphingomonas* and *Aspergillus*, *Bacilli*, *Pseudonocardia*, *Pantoea*, and *Jeotgalicoccus*. These microorganisms were thought to play a significant role in improving tobacco quality. *Bacillus* is crucial for regulating chemical composition, promoting accumulation of aroma compounds, and improving the quality of tobacco (Dai et al. 2020; Wu et al. 2021). *Pantoea* was the dominant microorganism for aging flue-cured tobacco surface (Su et al. 2011), which may be the reason why cigarettes and tobacco smoke have significant amounts of lipopolysaccharide. In the early phases of tobacco fermentation, *Jeotgalicoccus* may quickly metabolize reducing sugars and organic acids to influence tobacco aroma (Giacomo et al. 2007). The degradation products of terpenoids and higher fatty acids have been linked to *Aspergillus* (Wu et al. 2022).

Furthermore, Wu et al. (2022) screened *Aspergillus nidulans* strain F4 from tobacco and revealed that it secretes highly active lipoxygenase and peroxidase, which can increase the degradation of terpenoids and higher fatty acids to enhance tobacco flavor. Yu et al. (2011) also found that *Pseudomonas* was a dominant bacteria in nicotine degradation. Subsequent studies disclosed that *Pseudomonas* was capable of degrading nicotine, indicating that the pyrrolidine pathway facilitated this process (Tang et al. 2012).

## Future perspectives

Previous studies investigated only the composition and diversity of the microbial communities during the process of tobacco fermentation. To date, the functions and relevant metabolic pathways of individual microbial species during tobacco fermentation, and the effects of these relationships on chemical substances and flavonoids compounds, remain unclear. Nowadays, many advanced biotechnology-based approaches, sequencing techniques, and multi-omics technologies have become available. Future work could focus on this aspect. Besides, to produce higher-quality tobacco products, further research should focus on the potential use of functional strains as cultivation starters. In addition, stringent control of aging parameters represents a key strategy for ensuring consistent quality. By employing these approaches, the industry can unlock the potential for creating superior tobacco products with enhanced sensory characteristics and decreasing harmful substances.

## Conclusion

In conclusion, the aging process not only enhances certain health benefits but also affects the sensory properties of the tobacco product. Current research focuses primarily on the production of aroma compound and fermentation mechanisms of microorganisms based on multi-omics techniques. The main aroma metabolites of tobacco aging include organic acids, polyphenols, flavonoids compounds, amino acids and their derivatives. The pathways possibly involved in flavor formation of fermented foods are related to carbohydrate (EMP and glycolysis), citrate, amino acid, and fatty acid metabolism, indicated by a review of the literature. However, the influence of microorganisms on tobacco leaf color and combustion properties has not been extensively explored, thus presenting a potential avenue for future research. Besides, the current research focuses on the production of aroma and degradation of harmful substances.

## Data Availability

Data will be made available on request.
