# Correction: Changes in microbial composition during flue-cured tobacco aging and their effects on chemical composition: a review

**DOI:** 10.1186/s40643-025-00913-5

**Published:** 2025-07-04

**Authors:** Yangyang Yu, Yongfeng Yang, Tao Jia, Hang Zhou, Yao Qiu, Mengyao Sun, Hongli Chen

**Affiliations:** 1 College of Tobacco Science, Henan Agricultural University, No. 218, Pingan Road, Zhengdong-New District, Zhengzhou, 450002 China; 2 China Tobacco Henan Industrial Co., Ltd, Zhengzhou, 450000 China; 3 Tianchang International Co., Ltd, Xuchang, 461000 China

**Correction: Bioresources and Bioprocessing (2025) 12:43**

10.1186/s40643-025-00883-8

In this article the author’s name Tao Jia was incorrectly written as Jiatao Jia.

The original article has been corrected.

